# Non-Target Site Mechanisms Endow Resistance to Glyphosate in Saltmarsh Aster (*Aster squamatus*)

**DOI:** 10.3390/plants10091970

**Published:** 2021-09-21

**Authors:** José Alfredo Domínguez-Valenzuela, Ricardo Alcántara-de la Cruz, Candelario Palma-Bautista, José Guadalupe Vázquez-García, Hugo E. Cruz-Hipolito, Rafael De Prado

**Affiliations:** 1 Departamento de Parasitología Agrícola, Universidad Autónoma Chapingo, Texcoco 56230, Mexico; 2 Centro de Ciências da Natureza, Universidade Federal de São Carlos–Campus Lagoa do Sino, Buri 18290-000, Brazil; 3 Department of Agricultural Chemistry, Edaphology and Microbiology, University of Córdoba, 14014 Córdoba, Spain; z82pabac@uco.es (C.P.-B.); z82vagaj@uco.es (J.G.V.-G.); cruzhipolito@yahoo.com.mx (H.E.C.-H.); qe1pramr@uco.es (R.D.P.)

**Keywords:** aminomethylphosphonic acid, glyphosate metabolism, impaired translocation, phoenix phenomenon, reduced absorption

## Abstract

Of the six-glyphosate resistant weed species reported in Mexico, five were found in citrus groves. Here, the glyphosate susceptibility level and resistance mechanisms were evaluated in saltmarsh aster (*Aster squamatus*), a weed that also occurs in Mexican citrus groves. The R population accumulated 4.5-fold less shikimic acid than S population. S plants hardly survived at 125 g ae ha^−1^ while most of the R plants that were treated with 1000 g ae ha^−1^, which suffered a strong growth arrest, showed a vigorous regrowth from the third week after treatment. Further, 5-enolpyruvylshikimate-3-phosphate basal and enzymatic activities did not diverge between populations, suggesting the absence of target-site resistance mechanisms. At 96 h after treatment, R plants absorbed ~18% less glyphosate and maintained 63% of the ^14^C-glyphsoate absorbed in the treated leaf in comparison to S plants. R plants metabolized twice as much (72%) glyphosate to amino methyl phosphonic acid and glyoxylate as the S plants. Three non-target mechanisms, reduced absorption and translocation and increased metabolism, confer glyphosate resistance saltmarsh aster. This is the first case of glyphosate resistance recorded for *A. squamatus* in the world.

## 1. Introduction

Glyphosate is the active ingredient in numerous trade formulation herbicides for nonselective postemergence control of both annual and perennial weeds [1]. This herbicide disrupts the biosynthesis of tryptophan, phenylalanine and tyrosine by interrupting the activity of the enzyme 5-enolpyruvylshikimate-3-phosphate synthase (EPSPS) [2]. Glyphosate is used to control all types of annual and perennial weeds [3], being the main weed control tool in citrus groves in Veracruz, Mexico [4].

With more than 593,000 ha planted with citrus, mainly located in the state of Veracruz [5], Mexico is the fourth largest producer of citrus fruits worldwide [6], however, it is the main exporter of limes [7]. Citrus groves, particularly in the Gulf of Mexico, are subject to a heavy use of glyphosate [8], since rarely preemergent herbicides are used and other postemergence ingredients increase weed control costs [4]. As a result of basing weed control almost exclusively on glyphosate, some weed species evolved resistance to glyphosate. Of the six weed species reported to be resistant to glyphosate in Mexico, five were found in citrus groves [9].

Recently another weed species, common in citrus groves in the municipalities of Martinez de la Torre and San Rafael, Veracruz, has shown low levels of control with overdoses of 3–4 L ha^−1^ of commercial formulations of glyphosate (362 g ae L^−1^), considering that the recommended field dose is 720 g ae ha^−1^ [4]. Saltmarsh aster, *Aster squamatus* (Spreng.) Hieron. (Synonim: *Symphyotrichum squamatum* (Spreng) G. L. Nesom (Asteraceae), an annual weed native to South America but currently its distribution has extended to Asia, Europe, and North America [10,11], is one of the weed species that has not been efficiently controlled with glyphosate. Saltmarsh aster is found along the eastern coastal region of the Gulf Coast of Mexico [12], so it is a common weed occurring in citrus groves in the State of Veracruz. Seedling have broad leaves arranged in a rosette, and as the stem grows up, leaves tend to be narrow; plants height can be of up to 2 m [12].

For a better agricultural production in the presence of herbicide resistant weeds, it is essential to characterize the resistance mechanisms, since knowing such mechanisms contributes to implementing better weed management strategies. Fifty-one weeds have evolved resistance to glyphosate in the world [13], showing a great diversity of mechanisms endowing that resistance [14]. Replacement of amino acids at key positions in conserved regions of the EPSPS encoding gene, or EPSPS gene amplification inducing the overexpression of this enzyme are called target-site resistance (TSR) mechanisms. Physiological or metabolically responses such as the reduced absorption, impaired translocation, herbicide metabolism, vacuolar sequestration of glyphosate and, recently characterized foliar hypersensitivity that diminishes the amount of herbicide that reaches the EPSPS are called non-target site resistance (NTSR) mechanisms [15,16].

Saltmarsh aster has no official records of herbicide resistance in the International Herbicide-Resistant Weed Database [13], but it was reported with resistance to imazethapyr in Seville, Spain [17]. This present study was carried out to confirm the resistance to glyphosate in saltmarsh aster, a common weed in the citrus groves of Veracruz, Mexico, that has been poorly controlled with this herbicide in recent growing cycles, and to characterize its resistance mechanisms.

## 2. Results

### 2.1. Resistance Confirmation to Glyphosate 

**Figure 1:**
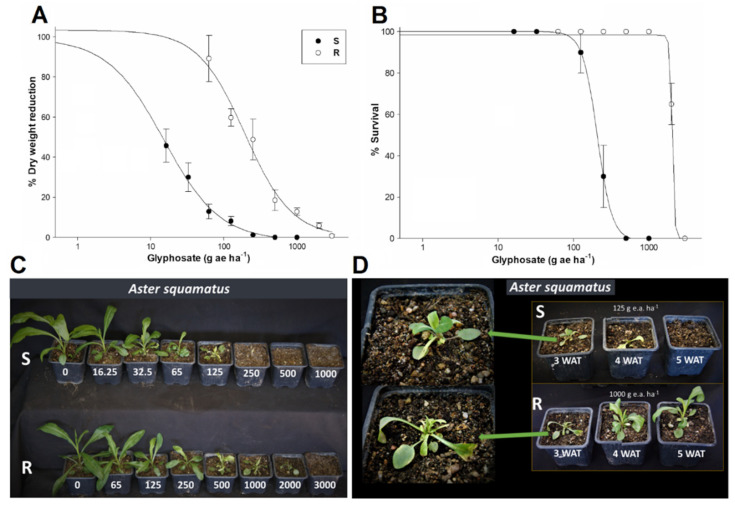
Glyphosate dose-response curves of the dry weight reduction (**A**) and plant survival (**B**) in susceptible (S) and resistant (R) saltmarsh aster (*A. squamatus*) populations treated with different glyphosate doses (g ae ha^−1^). Vertical bars are the SEM (*n* = 10 for weight reduction and *n* = 2 for plant survival). (**C**) R and S plants at 4 weeks after treatment (WAT) of glyphosate. (**D**) Regrowth of S and R plants treated with 125 and 1000 g ae ha^−1^ glyphosate, respectively, at different WATs.

Both saltmarsh aster populations showed high growth reduction showing low GR_50_ (herbicide rate that reduces plant growth by 50%) values (Figure 1A). The susceptible (S) population was highly susceptible to glyphosate (GR_50_ = 14.7 g ae ha^−1^); however, the resistant (R) population was 13-fold more resistant. The R population presented an LD_50_ (herbicide rate that kills 50% of the plant in a population) of 2078.1 g ae ha^−1^, which exceeded the field dose used in citrus groves (720 g ae ha−^1^), while all S plants died from glyphosate doses of 250 g ae ha^−1^. Based on the LD_50_ values, the R population presented a RF of 9.7 with respect to the S population (Figure 1B, Table 1). S plants hardly survived at 125 g ae ha^−1^; whereas most of the R plants survived up to 2000 g ae ha^−1^ (Figure 1C). R plants treated with 1000 g ae ha^−1^ suffered a growth arrest, but from 4 week after treatment displayed a vigorous regrowth (Figure 1D).

### 2.2. Acummulation of Shikimic Acid

**Figure 2:**
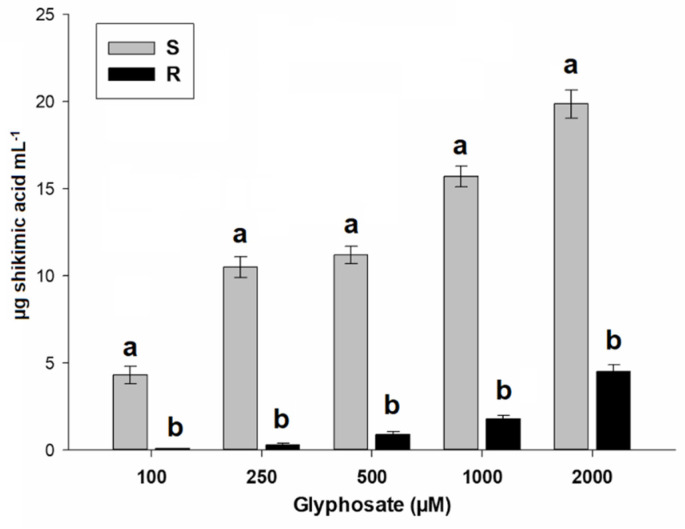
Accumulation of shikimic acid accumulation induced by glyphosate in susceptible (S) and resistant (R) saltmarsh aster populations (*A. squamatus*) found in Persian lime groves from Veracruz, Mexico. Vertical bars represent the standard error of the mean (*n* = 6). Different letter denotes differences between populations within a glyphosate by the Tukey test (*p* < 0.05).

The two saltmarsh aster populations presented shikimic acid accumulation, but the S population accumulated more shikimate than R population, regardless of glyphosate concentration. The accumulation ranged from 0.1 to 5 µg shikimic acid mL^−1^ as glyphosate concentration increased in the population R, while in the population S it went from 4 to 20.5 µg shikimic acid mL^−1^ (Figure 2).

### 2.3. Concentration and Activity of the EPSPS

**Figure 3:**
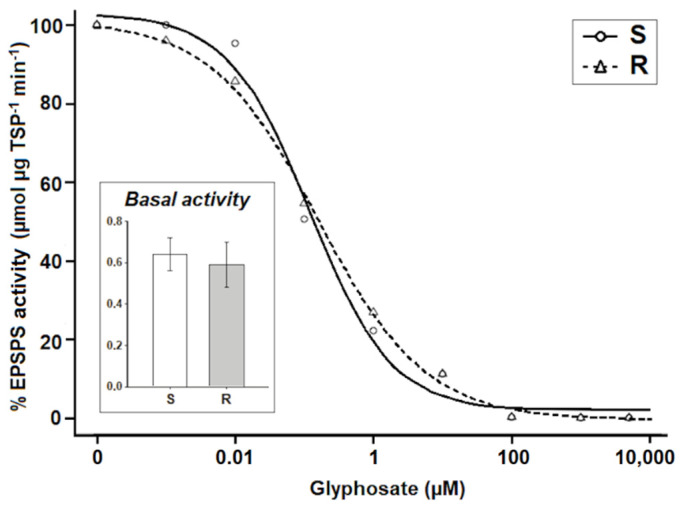
Enzyme activity of the 5-enolpyruvilshikimate-3-phosphate synthase (EPSPS) in glyphosate-susceptible (S) and -resistant (R) saltmarsh aster (*A. squamatus*) populations.

EPSPS basal and enzymatic activities of both saltmarsh aster populations showed no statistical differences. EPSPS activities of the S and R populations were 0.64 and 0.59 µmol of inorganic phosphate (Pi) released per µg of total soluble protein (TSP) per min (µmol Pi µg^−1^ TSP min^−1^), respectively, and only ~0.25 µM of glyphosate inhibited the EPSPS activity by 50% in both populations (Figure 3, Table 1).

### 2.4. Absorption and Translocation of ^14^C-Glyphosate

**Figure 4:**
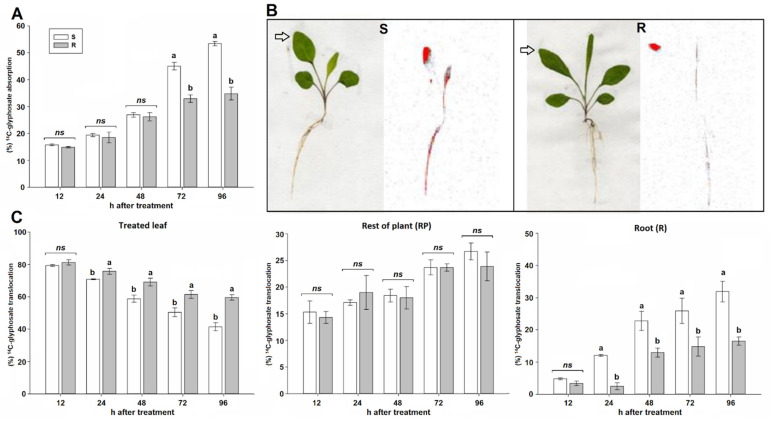
Absorption and translocation of ^14^C-glyphosate in susceptible (S) and resistant (R) saltmarsh aster (*A. squamatus*) populations from 12 to 96 h after treatment (HAT). (**A**) ^14^C-glyphosate absorption (% from the total applied). (**B**) Digital (on the left) and autoradiograph (right) images illustrating the ^14^C-glyphosate distribution within plants at 96 HAT. Red regions show higher signal (concentration) of ^14^C. (**C**) ^14^C-glyphosate translocation (% of the recovered) from the treated leaf to the rest of the plant, and roots. Vertical bars represent the standard error of the mean (*n* = 5). ns, no significant and different letter denotes differences between populations within an evaluation time by the Tukey test (*p* < 0.05).

The absorption of ^14^C-glyphosate increased from 15 to 53%, depending on the saltmarsh aster population, between 12 and 96 h after treatment (HAT). At short evaluation times there was no differences in absorption between populations, but from 72 HAT, population S absorbed between 12 and 18% more ^14^C-glyphosate than the R population, (Figure 4A), according to the ^14^C signals detected by liquid scintillation spectrometry (LSS). Qualitatively, a large amount of ^14^C from ^14^C-glyphosate remained in the treated leaves of the two populations, but the S one translocated more ^14^C to the roots than R population (Figure 4B). The population S translocated more ^14^C of the absorbed ^14^C-glyphosate to the remainder of the plant and root system than the R population from 24 HAT, and the differences increased over time, observing the greatest difference (18%) between populations at 96 HAT. The signals of ^14^C detected in the rest of the plant increased gradually from 14 to 27% with the passage of time, but did not differ between populations. However, the amount of ^14^C-herbicide that reached the roots, that ranged from to 4 to 32%, differed between populations from 24 HAT, always being higher in population S, and at 96 HAT, there was 15% more glyphosate in roots of S plants than in those of the R (Figure 4C).

### 2.5. Glyphosate Metabolism

Aminomethylphosphonic acid (AMPA) and glyoxylate are metabolites in which glyphosate can be metabolized by most higher plants [18]. Saltmarsh aster S and R plants metabolized up to 48 and 85% of glyphosate, respectively. The amounts of glyphosate, AMPA and glyoxylate found above-ground plant tissue ranged from 30 to 60, 4 to 37 and 4 to 39 nmol g^−1^ fresh weight, respectively, depending on the population and evaluation time. The S plants transformed between 7 and 19% of the glyphosate into AMPA and between 6 and 17% in glyoxylate from 48 to 96 HAT; while R plants metabolized between 18 and 35% in AMPA, and from 17 to 37% in glyoxylate over time (Table 2).

## 3. Discussion

The S and R saltmarsh aster populations from Mexico showed contrasting susceptibility levels to glyphosate. Plants of population R had a 50% of weight reduction with 200 g ae ha^−1^, but most of them survived at doses of up to 2000 g ae ha^−1^, which is a glyphosate concentration well above the field dose of 720 g ae ha^−1^ widely used in citrus groves of the Gulf Coast of Mexico [4]. This plant survival rate confirms farmers complaints about poor control of saltmarsh aster with glyphosate, supporting the hypothesis of selection of resistant individuals. In addition, resistance ratios were 13.7 and 9.7 with respect to weight reduction and plant survival, respectively. S population of another Asteraceae weed species found in citrus groves in the same area of the Gulf Coast of Mexico, such as *Bidens pilosa* and *Parthenium hysterophorus*, were greatly sensitive to glyphosate (GR_50_ values of 52 and 43 g ae ha^−1^, respectively), while some R plants of both species also survived at doses higher that 2000 g ae ha^−1^ [19,20].

In addition to poor saltmarsh aster control, farmers also reported a vigorous regrowth of the treated plants starting in the third week after glyphosate had apparently compromised their development. That field observation was corroborated in this study, where S plants treated with 125 g ae ha^−1^ died or hardly survived, while R plants survived and regrew even at 2000 g ae ha^−1^. Regrowth was previously documented for glyphosate-resistant *Ambrosia trifida*, another Asteraceae weed [21,22]. However, different to saltmarsh aster, giant ragweed presented a rapid necrosis and death of leaf tissue in the first 48 HAT followed by vigorous regrowth and, at 3 WAT, the treated R plants already had a normal appearance [16], for which this regrowth was called “phoenix phenomenon” [21]. Similar to *A. trifida*, *Conyza sumatrensis* from Brazil also presented rapid necrosis followed by a regrowth after 2,4-D treatment [23]. In both species, the mechanism that induced rapid necrosis is not fully understood, but it was suggested that cell death was due to the high production and action of reactive oxygen species (ROS), preventing the translocation of herbicides to apical growing meristems [21,23]. ROS production levels were not determined in saltmarsh aster populations, and due to the longer time that R plants took to restart growth, it is unlikely that the phoenix phenomenon was responsible for regrowth in this population. Additional studies are required to characterize the mechanisms that enable vigorous regrowth of R saltmarsh aster plants.

The accumulation of shikimic acid, widely used as a bioindicator of the level of susceptibility to glyphosate [24], confirmed the resistance of the R saltmarsh aster population that accumulated 4.5 times less shikimate than the S population, showing little or limited interaction of the glyphosate with the EPSPS [25]. However, shikimic acid accumulation tests do not reveal which type of TSR or NTSR mechanism confer resistance [26]. In this sense, enzymatic activity assays of herbicide target enzymes are important to reveal a possible involvement of TSR mechanisms [27]. However, TSR mechanisms such as EPSPS mutations or EPSPS gene amplification was ruled out in the R saltmarsh aster population, because their EPSPS basal and enzymatic activities did not diverge with respect to the S population. Thus, mechanism (s) endowing resistance to glyphosate in the population R was restricted to NTSR.

Absorption and translocation of ^14^C-glyphosate by R saltmarsh aster plants was lower than in S saltmarsh aster plants. Foliar herbicide absorption differences between R and S plants have been linked to anatomical characteristics of the leaves [14], such as leaf cuticle, trichome distribution, growth stage and leaf structure [28,29]. Reduced absorption is a rare herbicide resistance mechanism and has been documented in several weed species resistant to glyphosate such as *Amaranthus tuberculatus* [30], *Lolium multiflorum* [31], *Sorghum halepense* [32], to mention some cases, as well as to other herbicides [33]. However, how plants diminish herbicide absorption rates is not fully understood. The R saltmarsh aster plants absorbed less glyphosate than S plants, which somehow allows them to survive higher doses of herbicide.

Systemic herbicides such as glyphosate must be translocated to apical growing meristems to reach its target site in sufficient amounts to act and be lethal to weeds [34]. Given that the movement of ^14^C-glyphosate from the treated leaves to the rest of the plants and roots was limited for the R saltmarsh aster plants, adding to this that this population also absorbed less herbicide, it can be assumed that the amount of glyphosate that reached the EPSPS was not enough to completely inhibit it. Reduced translocation may occur when glyphosate is compartmentalized into vacuoles or trichomes of leaves close to the area where it was applied, or by altered activity of active membrane transporters [16,35]. Among the NTSR mechanisms, reduced glyphosate translocation is the most reported mechanism endowing resistance to this herbicide [30,32,36,37], which appears to be regulated by a single incompletely dominant nuclear gene [38]. Moreover, reduced absorption and translocation of ^14^C-glyphosate sometimes work in concert [16]; therefore, these two NTSR mechanisms contributed to the overall resistance of the R saltmarsh aster plants. At least one population of every glyphosate resistant weed species found in citrus groves from Mexico showed some of these NTSR mechanisms [19,20,26,39,40].

The population R of saltmarsh aster metabolized a greater amount of glyphosate in less time than the population S. Glyphosate metabolism as a resistance mechanism was first reported in *Digitaria insularis* determined by analytical techniques [41]. This finding generated controversy for not demonstrating its occurrence through molecular studies [20]. In addition, previously it had been concluded that weeds were unlikely to degrade glyphosate [42]. However, the enzyme aldo-keto reductase from glyphosate-resistant *Echinochloa colona* was recently found, following analytical and molecular approaches, capable of metabolizing glyphosate into AMPA and glyoxylate [43]. Although the work of Pan et al. [43] proved that metabolism may contribute in the resistance to glyphosate, it is presumed that this is not the main resistance mechanism in *E. colona* [18,44], since both R and S populations metabolized the herbicide. This observation is based on the fact that the transgenic canola plants to which the bacterial GOX (glyphosate oxidoreductase) gene was inserted, although they metabolize glyphosate, it was not enough to survive field applications [41,43]. Although the S population of *E. colona* metabolized 44% of the glyphosate, while the R population up to 90% of the herbicide at 72 HAT [43], McElroy and Hall [44] suggested that resistance in *E. colona* is based on a TSR mechanism (Pro-106-Thr mutation), which was obfuscated because *E. colona* is a polyploid species. In the case of saltmarsh aster, the S and R populations metabolized 27 and 50% of glyphosate, respectively, at 72 HAT, and up to 36 and 72% at 96 HAT, i.e., glyphosate metabolism was lower and slower than in *E. colona*. Therefore, this mechanism possibly contributes to the glyphosate resistance of the R saltmarsh aster population, but it is difficult to measure the magnitude of its contribution or to affirm that it performs a significant role [42]. However, it is necessary to deepen studies on candidate enzyme genes involved in the metabolism of this herbicide, since it seems that this mechanism is capable of conferring resistance to glyphosate [45].

## 4. Materials and Methods

### 4.1. Biological Material

Mature seeds of the R saltmarsh aster population were harvested in a Persian lime grove in the municipality of San Rafael, Veracruz, Mexico (20°06′28″ N, 97°09′34″ W) from were collected from surviving adult plants (minimum 50) in the field at the last application of glyphosate at the recommended time and rate (720 g ae ha^−1^). Lime trees had a 15-year history of continuous glyphosate use (3–4 applications per year). Seeds from a never-treated population (S) were collected from a nearby grove where the R population was also obtained. The pool of seeds from an orchard constituted a sample of a single R and S population. 

The seeds were sown in trays (15 × 15 × 8 cm) on wet substrate (peat at field capacity) hermetically closed until germination and placed in a growth chamber at 26/18 °C (day/night), 16-h photoperiod at 850 μmol^−2^s^−1^ of light intensity and 60% of RH. Seedlings with two true leaves were individualized into 250-mL punnet pots containing sand/peat (1:1, *v*/*v*) fertilized with 400 mg of NPK 17-09-11 + 2% MgO. The pots were maintained in the growth chamber conserved the growing conditions until herbicide treatments. Plants were watered daily or as needed.

### 4.2. Dose-Response Curves

The tests were conducted on whole plants with 4 to 6 true leaves of the R and S populations. The doses of glyphosate (Roundup Energy 450 g L^−1^, Bayer Spain) sprayed on the population S were 16.125, 31.25, 62.5, 125, 250, 500 and 1000 g ae ha^−1^, while for the population R they were 65, 125, 250, 500, 1000, 2000 and 3000 g ae ha^−1^. Ten plants taken randomly from the corresponding population were treated per herbicide dose. Spraying was performed in an herbicide spray cabinet (SBS-060 De Vries Manufacturing, Hollandale, MN, United States). The cabinet application bar had an XR8002E tip and was calibrated to spray 200 L ha^−1^ at a pressure of 250 kPa.

Four weeks after treatment (WAT), the aerial plant tissue was cut at ground level, conditioned in and identified in paper envelopes, and dried for 4 days at 60 °C. Then, samples were weighed and transformed into dry weight percentage to the GR_50_ with respect to the untreated control. Additionally, plant mortality per dose was assessed to determine LD_50_ values. The experiments were repeated twice, once in June 2019 and the second in January 2020.

### 4.3. Shikimic Acid Accumulation

For each population of saltmarsh aster, approximately 25 fully extended young leaves were taken from at least 15 plants with 4 to 6 true leaves and 4-mm diameter discs were cut. Samples of 50 mg leaf discs (pool foliar tissue) were placed in 2-mL tubes containing 1 mL of 10-mM ammonium phosphate monobasic solution (pH adjusted to 4.4 with 0.1 HCl) at different rates of glyphosate (0, 100, 250, 500, 1000, and 2000 µM). The extraction of shikimic acid and quantification was performed according to Shaner et al. [24] with small adaptations [46]. Three samples of each saltmarsh aster population were assessed per glyphosate concentration. The experiment was completely randomized and was repeated twice. Results were expressed as micrograms of shikimate per mL HCl solution (µg shikimic acid mL^−1^) in relation to a calibration curve of shikimic acid.

### 4.4. Enzymatic Activity of EPSPS

Samples of 10-g fresh leaf tissue of each saltmarsh aster population were collected and immediately macerated in liquid N_2_ until obtaining a fine powder. After an extraction step, following the procedures detailed by Dayan et al. [27], samples were dialyzed in a 1000-MWC dialysis tubing (30 mm) at 4 °C on a stir plate over 12 h. The protein concentration (basal activity) without glyphosate was determined by the Bradford method. Then, the EPSPS enzymatic activity of the S and R populations was assayed using a phosphate assay kit (EnzCheck, Invitrogen, Carlsbad, CA, USA) according to the manufacturer guidelines. Phosphoenolpyruvate (1.02 mM) and shikimate-3-phosphate (0.41 mM) were the substrates to check EPSPS activities under different glyphosate concentrations (0, 0.001, 0.1, 1, 10, 100, 1000 and 5000 μM). The EPSPS activity was determined by measuring the amount (µmol) of Pi released per µg of TSP per min during 10 min at 360 nm in a spectrophotometer [26]. These tests were repeated twice evaluating three samples by glyphosate concentration and population, and for each sample three technical replicates were evaluated. The rate of glyphosate required to inhibit the EPSPS activity by 50% (I_50_) was determined.

### 4.5. ^14^C-Glyphosate Absorption and Translocation

^14^C-glyphosate [glycine-2-^14^C, specific activity 273.8 MBq mmol^−1^, Institute of Isotopes Co. Ltd. Budapest, Hungary) was mixed with commercially formulated glyphosate. The specific activity was of the radiolabeled solution was 25,000 dpm µL^−1^ (=417 Bq µL^−1^), and the rate of glyphosate was 720 g ae ha^−1^ in 200 L ha^−1^. The second leaf was identified and protected with aluminum film. After this, plants were treated with glyphosate (720 g ae ha^−1^), and 30 min later, the aluminum layer was removed [47]. The labeled herbicide solution was applied to the adaxial surface of the second leaf, that had been protected with aluminum layer, of 25 plants of each saltmarsh aster population in four 0.5 µL droplets with a PB-600 repeating dispenser (Hamilton Company, Reno, NV, USA), i.e., each plant received a total of 50,000 dpm. The treated area of R and S populations was washed in sets of three plants at different intervals (12, 24, 48, 72, and 96 HAT) to recover the unabsorbed ^14^C-glyphosate with 1.5 mL of acetone three times each plant. Three mL liquid scintillation cocktail (Ultima Gold, Perkin-Elmer, BV BioScience Packard, Waltham, MA, USA) was added to each wash sample and analyzed by LSS for 10 min. 

To determine the translocation rates of ^14^C-glyphosate, the whole plants were removed from the punnet pots, and the roots were carefully washed and dried and fixed on paper. Plants were dried at 60 °C for 4 d and then placed adjacent to a phosphorus storage film (Storage Phosphor System: Cyclone, Perkin-Elmer Packard BioScience BV) for 10 h in the dark. ^14^C-glyphosate distribution within plants was scanned in a phosphor storage system (Cyclone Plus, Perkin-Elmer). Then, plants were sectioned into treated leaves, the rest of the plants, and roots and burned in a sample oxidizer (Packard 307) for 3 min. Released ^14^CO_2_ was captured in 14 mL of Carbosorb/Permafluor E+ (7/7 *v*/*v*; Perkin-Elmer), and radioactivity was quantified by LSS by 10 min. Mass balance (recovery percentage of ^14^C-glyphosate) and the rates of absorption (from total applied) and translocation (from recovered) were calculated according to Alcántara-de la Cruz et al. [34].

### 4.6. Glyphosate Metabolism

Saltmarsh aster plants at the 4–6 leaf stage of both populations were treated with 720 g ae ha^−1^ under the spraying conditions used in “dose-response curves”. At 48, 72 and 96 HAT, the above ground tissue was harvested, washed, and macerated in liquid N_2_ and prepared for extraction according to Rojano-Delgado et al. [48]. The extracted samples were analyzed by reversed polarity capillary electrophoresis in a 3D Capillary Electrophoresis Agilent G1600 equipped with a diode array detector (DAD, wavelength range 190–600 nm), using a background electrolyte of 10 mM potassium phthalate, 0.5 mM hexadecyltrimethylammonium bromide (CTAB) and 10% acetonitrile at pH 7.5. Calibration curves of glyphosate, AMPA and glyoxylate of analytical grade (purity < 95%, Sigma-Aldrich, Spain) were used for their quantification. In addition, the glyoxylate naturally produced by the plants was measured in nontreated plants and subtracted from the average found in glyphosate treated plants [48], i.e., the amount of glyoxylate reported correspond to that presumably from glyphosate metabolism. The experiment was repeated two times, with five replicates per population and evaluation time.

### 4.7. Statistical Analysis

The GR_50_, LD_50_ and I_50_ values of each saltmarsh aster population were determined by submitting the percentage data of dry weight reduction, plant survival and enzyme activity, respectively, to a nonlinear regression analysis using the three-parameter log-logistic model: Y = (*d*/[1 + (*x*/*g*)*^b^*]) [49]. Regression analyses were performed on SigmaPlot 11. After this, the resistance factors (RF = R/S) of each parameter (GR_50_, LD_50_ or I_50_) were calculated. 

Data obtained from the shikimic acid accumulation, EPSPS basal activity, absorption, translocation and metabolism of glyphosate were analyzed by one-way ANOVA. When necessary, Tukey’s HSD test (95% confidence level) was used to separate the means.

## 5. Conclusions

This study confirmed glyphosate resistance in saltmarsh aster collected in citrus groves from the Gulf Coast of Mexico. Resistance is endowed by three non-target site mechanisms: reduced absorption, impaired translocation and metabolism of glyphosate. The combination of these mechanisms allows R aster individuals to survive and regrow vigorously after having suffered a severe growth arrest caused by glyphosate. The results of this work report the first case of glyphosate resistance in *Aster squamatus*, and it is the sixth species to evolve resistance to this herbicide in citrus groves from Mexico.

## Figures and Tables

**Table 1:** Parameters of the three-parameter log-logistic equation ^a^ used to estimate the weight reduction, plant mortality or enzyme activity inhibition by 50% in glyphosate-susceptive (S) and -resistant (R) saltmarsh aster populations.

Dose-Response	Population	*b*	*d*	*g*	RF
Weight reduction (g ae ha^−1^)	S	1.2	99.9	14.7 ± 1.2	13.7
	R	1.3	101.1	202.1 ± 17.2	
Plant mortality (g ae ha^−1^)	S	5.5	100.0	214.3 ± 11.4	9.7
	R	7.1	99.9	2078.1 ± 60.9	
Enzyme inhibition (µM)	S	0.2	99.6	0.27 ± 0.04	0.85
	R	4.2	100.5	0.23 ± 0.02	

^a^ Y = *d*/[1 + (*x*/*g*)*^b^*], where Y is the percentage weight reduction, plant mortality or enzyme activity in comparison to the respective nontreated control, *d* is the upper asymptote of the curve, *b* is the slope of the curve, *g* is the inflection point of the curve halfway (GR_50_, LD_50_ or I_50_), and x is the herbicide dose tested. Resistance factors (RF = R/S) are the R-to-S LD_50_, ED_50_ or I_50_ ratios.

**Table 2:** Amount of glyphosate and metabolites (nmol g^−1^ fresh weight) in resistant (R) and susceptible (S) saltmarsh aster populations at different hours after treatment (HAT) with 720 g ae ha^−1^ of glyphosate.

HAT	Population	Glyphosate	AMPA *	Glyoxylate
48	S	53.3 ± 4.2 (87) a	4.4 ± 0.8 (7) b	3.9 ± 0.6 (6) b
	R	45.5 ± 3.8 (65) b	12.6 ± 2.0 (18) a	11.7 ± 1.9 (17) a
72	S	57.7 ± 4.7 (73) a	11.2 ± 1.4 (14) b	10.1 ± 1.2 (13) b
	R	43.6 ± 3.6 (50) b	22.3 ± 2.2 (26) a	21.1 ± 3.1 (24) a
96	S	59.8 ± 4.6 (64) a	18.1 ± 1.8 (19) b	16.3 ± 2.3 (17) b
	R	30.1 ± 2.6 (28) b	37.3 ± 4.1 (35) a	39.8 ± 4.3 (37) a

* Aminomethylphosphonic acid. Means ± SE (relative %). Different letter denotes differences between populations within an evaluation time by the Tukey test (*p* < 0.05).

## Data Availability

Not applicable.
